# Percutaneous Discectomy—Continuous Irrigation and Drainage for Tuberculous Lumbar Spondylitis: A Report of Two Cases

**DOI:** 10.1155/2009/632981

**Published:** 2009-11-08

**Authors:** Sei Shibuya, Satoshi Komatsubara, Tetsuji Yamamoto, Nobuo Arima, Yoshiaki Kanda, Shiro Oka

**Affiliations:** ^1^Department of Orthopaedic Surgery, Kagawa University School of Medicine, Kagawa 761-0793, Japan; ^2^Oka Orthopaedic and Rehabilitation Clinic, Kagawa, Japan

## Abstract

Percutaneous curettage and continuous irrigation were performed for definitive diagnosis and treatment of tuberculous (TB) lumbar spondylitis. Under local anaesthesia, affected lumbar discs were curetted using a procedure of percutaneous nucleotomy, and in-tube and the out-tube were placed for continuous irrigation. The period of continuous irrigation was 12–16 days. *Mycobacterium tuberculosis* was demonstrated in case 1 by culture and PCR, whereas histology showed tuberculous lesion with caseous necrosis in both cases. Postoperative MRI showed markedly reduced abscesses after 3 months in both cases. The signal intensity in vertebral bodies was improved. In Case 2, CT observations showed remodeling over time in the vertebral body cavities. This method is advantageous in that although minimally invasive, it achieves identification of pathogenic bacteria and treatment simultaneously. This surgical procedure is expected to prove effective for both TB and pyogenic spondylitis.

## 1. Introduction

Since tuberculous (TB) spondylitis does not induce strong acute inflammation such as that observed for pyogenic spondylitis, patients are unlikely to visit a clinic immediately after onset and tend to be diagnosed after progression of bone destruction and abscess formation, even if neurological dysfunction has not yet developed [1].

As for treatment, conservative therapy using antituberculous agents has been used for early-stage disease without nerve paralysis and with little destruction of vertebral bodies, while surgical therapy aimed at focal resection and spinal reconstruction has been performed in cases displaying advanced bone destruction combined with paralysis [2].

Recently, as an application of minimally invasive percutaneous nucleotomy (PN) under local anesthesia [3], good surgical outcomes have been reported for pyogenic spondylitis using percutaneous curettage and continuous irrigation [4, 5]. The present report describes the good results obtained using this technique of PN for definitive diagnosis (histological and microbiological analysis) and treatment in 2 cases of TB lumbar spondylitis.

## 2. Case Reports

**Case 1**

A 74-year-old woman with a history of gastric cancer presented with a chief complaint of lower back pain. Subjective lower back pain emerged approximately 4 months prior to her visit to our institute.

On admission, deep tendon reflexes in both legs (patella tendon reflex (PTR) and Achilles tendon reflex (ATR)) were found to be decreased and hypoaesthesia was identified in the left L4 sensory region. Muscle weakness was observed in the left tibialis anterior muscle according to the manual muscle test (MMT), with a score of 4. Mild perianal hypoesthesia was observed. Neurologically, cauda equina syndrome and radiculopathy at and below the L4 level were identified.

**Figure 1:**
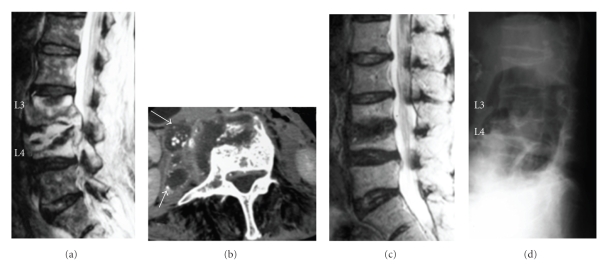
(a) Preoperative MRI in Case 1: T2-weighted imaging. Abscess formation with signal hyperintensity is observed between vertebral bodies and epidurally. (b) Preoperative contrast-enhanced CT. Abscess formation (arrow) is visible around the vertebral bodies, with calcification inside. (c) T2-weighted imaging at 18 months postoperatively. Resolution of abscess is indicated. (d) Plain radiography at 30 months postoperatively. Complete bone union has been achieved.

Regarding magnetic resonance imaging (MRI), T1-weighted imaging showed overall signal hypointensity for both L3 and L4 vertebral bodies, although T2-weighted imaging showed signal hyperintensity presumably indicating abundant abscess accumulation in the disc space, a lesion suspected to be epidural abscess in the spinal canal, and marked compression of the dural sac (Figure 1(a)). Contrast-enhanced computed tomography (CT) showed multiple abscess formation around vertebral bodies with calcification inside (Figure 1(b)). The preoperative diagnosis was infectious spondylitis (TB or pyogenic).

Surgery was started with the patient in the lateral position under local anaesthesia. According to the PN manoeuvre, percutaneous puncture was performed into the L3/4 intervertebral disc. A sheath was inserted into the disc space to aspirate 30 mL of abscess, and lower back pain reduced immediately thereafter. Curettage was performed while checking anteroposterior and lateral views on an X-ray image intensifier. Finally, an epidural tube as the in-tube and a drainage tube of 3 mm diameter as the out-tube were indwelled in the disc space and the operation was completed. Continuous irrigation was then started.

On postoperative day 3, *Mycobacterium tuberculosis* was detected as Gaffky grade 2. *M. tuberculosis* infection was also confirmed by tuberculosis-polymerase chain reaction (TB-PCR). Irrigation was thus continued until postoperative day 16 using a washing solution of 200 mL saline containing 1000 mg streptomycin each day. Multidrug antituberculous therapy was started using pyramide (900 mg/d), isoniazid (300 mg/d), rifampicin (300 mg/d), and ethambutol (500 mg/d). Immediately after the completion of irrigation, the patient wore a lumbar brace and started rehabilitation of walking and muscle strength of the legs. Histology showed typical TB tissue with central caseous necrosis, surrounded by epithelioid cells and Langhans-type giant cells. Severe lower back pain also resolved almost completely by 1-2 weeks postoperatively.

MRI at 3 months postoperatively showed a clear tendency toward recovery of normal signal intensity in the L3 and L4 vertebral bodies on T1-weighted imaging, while most of the abscess with high signal intensity had disappeared from T2-weighted imaging. Good improvements were seen in both diagnostic imaging and clinical symptoms, with no tendency toward recurrence in blood biochemistry. All antituberculous agents were therefore discontinued at 7 months postoperatively. On MRI at 18 months postoperatively, signal intensity in vertebral bodies had completely recovered on T1-weighted imaging. No signal hyperintense regions were apparent in the disc space or epidurally to suggest abscess recurrence on T2-weighted imaging (Figure 1(c)). In addition, plain radiography indicated complete bone union at 30 months postoperatively (Figure 1(d)). At present, 7 years and 6 months after surgery, follow-up has shown good condition with no relapse of symptoms.

**Case 2**

A 64-year-old woman was referred to our hospital with lower back pain and a history of surgery for leg varicose veins. Lower back pain had emerged about 3.5 months prior to her visit to our institute, with gradual worsening. The patient complained that lower back pain had never improved since onset and had been exacerbated by even slight exercise such as standing and walking.

**Figure 2:**
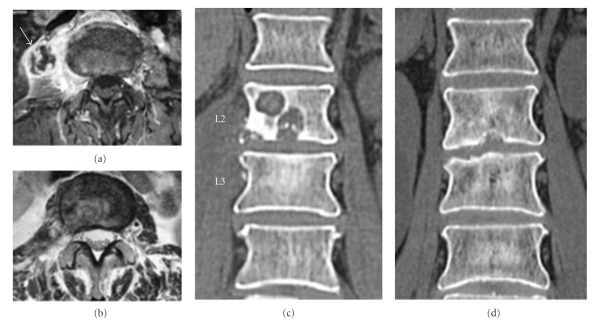
(a) Preoperative gadolinium imaging, axial image. Psoas abscess formation is visible (arrow), along with rim enhancement. (b) T2-weighted imaging at 3 months postoperatively, axial image. The iliopsoas abscess has disappeared. (c) Preoperative CT shows clear cavitations within the L2 vertebral body. (d) CT at 12 months postoperatively indicates that the cavity within the vertebral body has disappeared and has been remodeled.

Regarding MRI, T1-weighted imaging showed overall signal hypointensity in both L2 and L3 vertebral bodies, whereas T2-weighted imaging showed signal hyperintensity presumably indicating abscess accumulation in the disc space extending to inside the L2 vertebral body. Subsequent gadolinium-enhanced imaging showed diffuse contrast in both L2 and L3 vertebral bodies and rim enhancement around the abscess in the right psoas muscle (Figure 2(a)). CT showed a destructive change in the L2 vertebral endplate, and cavitations ≥10 mm in diameter were found in vertebral bodies (Figure 2(c)). Preoperative diagnosis was infectious spondylitis (TB or pyogenic).

Surgery was performed similar to that described for Case 1. A sheath was inserted into the L2/3 disc space to allow curettage of infected disc tissue. Finally, a 3 mm diameter in-tube and a 5 mm diameter drainage tube as the out-tube were indwelled in the disc space and the operation was completed. Continuous irrigation was started.

Histology on postoperative day 9 showed epithelioid cells centred around caseous necrosis, and QuantiFERON TB-2G showed strongly positive results. TB spondylitis was therefore finally diagnosed. Irrigation was performed until postoperative day 12 using only 250 mL of saline each day. Multidrug antituberculous therapy was started using isoniazid (300 mg/d), rifampicin (450 mg/d) and ethambutol (750 mg/d). After completion of irrigation, the patient wore a lumbar brace and started rehabilitation, mainly for walking. Strong lower back pain also disappeared by about 1-2 weeks postoperatively.

MRI at 3 months postoperatively showed recovery to normal signal intensity from signal hypointensity in the L2 and L3 vertebral bodies, except for the cavity area on T1-weighted imaging. On T2-weighted imaging, part of the signal hyperintense area remained, but was restricted to the disc space and vertebral bodies, with disappearance of abscess in the right iliopsoas muscle (Figure 2(b)). By 6 months postoperatively, diagnostic imaging showed good improvement, as did clinical symptoms. There was no evidence of recurrence from blood biochemistry. Administration of all antituberculous agents was thus discontinued at 6 months postoperatively. At 12 months, signal intensity in L2 and L3 vertebral bodies had normalized on T1-weighted imaging. No signal hyperintense regions were seen in the disc space, inside vertebral bodies or in the psoas muscle on T2-weighted imaging.

By 6 months postoperatively, CT showed repair of the L2 vertebral body cavity and bone remodelling, and the cavities was clearly reduced. At 12 months postoperatively, the cavity was completely repaired and disappeared (Figure 2(d)). At the time of writing, although complete bone union has not been achieved, follow-up shows good condition without relapse of symptoms at 24 months postoperatively.

## 3. Discussion

TB spondylitis resistant to conservative therapy is treated surgically by focal dissection of sequestrum and abscess, followed by spinal reconstruction using bone graft [2]. As less biofilm formation by *M. tuberculosis* is seen around metal implants than by other pyogenic bacteria [6], surgical treatment in combination with spinal instrumentation is increasingly considered acceptable. Reported methods include support by posterior instrumentation after curettage of the anterior parts of vertebral bodies and bone graft [7] and reconstruction using anterior instrumentation after curettage and bone graft [8]. A method to obtain strong stability has also been reported using not only bone graft but also a titanium cage for reconstruction of vertebral bodies [9]. These methods have recently been applied to pyogenic spondylitis but are definitely highly invasive surgeries, either as 1- or 2-stage procedure.

Conversely, a method of percutaneous surgery for pyogenic spondylitis is available as an alternative to conservative treatment with antibiotic administration or open surgery. In 1990, Onik et al. reported 3 cases of automated percutaneous discectomy for the purpose of biopsy [10]. Yu et al. provided the first report of percutaneous discectomy in 2 cases of lumbar osteomyelitis using a nucleotome in 1991 [11]. Nagata et al. later reported that good results were obtained through continuous irrigation with 2 indwelled tubes (an irrigation tube and drainage tube) in addition to percutaneous discectomy and local irrigation [4].

Other methods have also been reported to obtain good results, including external fixation in combination with percutaneous surgery [12], secure local debridement using an endoscope [13], and abscess drainage and continuous irrigation, even in cases with marked abscess and bone destruction [5]. Most reports of percutaneous discectomy have involved continuous irrigation and drainage of pyogenic spondylodiscitis. No reports have described advanced TB spondylitis.

Dinc et al*.* performed CT-guided percutaneous drainage in 21 cases of TB psoas and spondylodiscitic abscess, with periods of drainage of up to 36 days [14]. However, 6 of these cases showed recurrence after removal of the drainage catheter. In a report of percutaneous discectomy in 16 cases of lumbar spondylodiscitis (including 1 case of TB), the primary objective was definitive diagnosis by focal biopsy. Three cases later required interbody fusion by open surgery [15].

Surgical methods for TB spondylitis with marked abscess formation and bone destruction have been thought to require direct pus drainage, focal resection, and spinal reconstruction by bone graft. However, for patients with no advanced motor palsy before surgery, and no severe kyphotic deformity associated with vertebral body destruction or severe spinal instability, the method described herein can be expected to be effective not only for pyogenic spondylitis but also for TB spondylitis.
